# Protein methylation is required to maintain optimal HIV-1 infectivity

**DOI:** 10.1186/1742-4690-3-92

**Published:** 2006-12-15

**Authors:** Nicole M Willemsen, Eleanor M Hitchen, Tracey J Bodetti, Ann Apolloni, David Warrilow, Sabine C Piller, David Harrich

**Affiliations:** 1 Division of Immunology and Infectious Disease, Queensland Institute of Medical Research, Brisbane, Queensland, 4006, Australia; 2 HIV Protein Functions and Interactions Group, Centre for Virus Research, Westmead Millennium Institute, Westmead NSW 2145, Australia

## Abstract

**Background:**

Protein methylation is recognized as a major protein modification pathway regulating diverse cellular events such as protein trafficking, transcription, and signal transduction. More recently, protein arginine methyltransferase activity has been shown to regulate HIV-1 transcription via Tat. In this study, adenosine periodate (AdOx) was used to globally inhibit protein methyltransferase activity so that the effect of protein methylation on HIV-1 infectivity could be assessed.

**Results:**

Two cell culture models were used: HIV-1-infected CEM T-cells and HEK293T cells transfected with a proviral DNA plasmid. In both models, AdOx treatment of cells increased the levels of virion in culture supernatant. However, these viruses had increased levels of unprocessed or partially processed Gag-Pol, significantly increased diameter, and displayed reduced infectivity in a MAGI X4 assay. AdOx reduced infectivity equally in both dividing and non-dividing cells. However, infectivity was further reduced if Vpr was deleted suggesting virion proteins, other than Vpr, were affected by protein methylation. Endogenous reverse transcription was not inhibited in AdOx-treated HIV-1, and infectivity could be restored by pseudotyping HIV with VSV-G envelope protein. These experiments suggest that AdOx affects an early event between receptor binding and uncoating, but not reverse transcription.

**Conclusion:**

Overall, we have shown for the first time that protein methylation contributes towards maximal virus infectivity. Furthermore, our results also indicate that protein methylation regulates HIV-1 infectivity in a complex manner most likely involving the methylation of multiple viral or cellular proteins and/or multiple steps of replication.

## Background

Protein methylation is a post-translational modification by which a methyl group from S-adenosylmethionine is added to a protein. In eukaryotes, proteins can be methylated on the side chain nitrogens of arginine, lysine, and histidine residues or on the carboxyl groups of proteins [1]. Methylation on side chain nitrogens is considered largely irreversible while methylation of the carboxyl groups is potentially reversible [2]. Peptidylarginine deiminase activity can remove some methyl groups from methylated arginine forming a non-charged citruline residue [3-5]. Similar to other post-translational modifications, protein methylation is involved in regulating protein-protein interactions resulting in a plethora of effects during key cellular events, including regulation of transcription [6-8], stress response, ageing and protein repair [9], T-cell activation [10], nuclear transport [11], neuronal differentiation [12,13], ion channel function [14,15], and cytokine signaling [16].

The recent discovery of the enzyme family of the protein arginine methyltransferases (PRMTs), as well as technical advances that allow the specific detection of methylated proteins [17,18] have made PRMTs of particular interest. There are different PRMT isoforms that possess four types of activities which transfer methyl groups from S-adenosyl-L-methionine (AdoMet) to the guanidino group of arginine residues [reviewed in [19]]. PRMTs can modify arginine residues by adding one or two methyl groups resulting in three distinct forms of methylated arginine residues in eukaryotes, ω-N^G^-monomethylarginine (MMA), asymmetric (a) and symmetric (s) ω-N^G^, N^G^-dimethylarginine (aDMA and sDMA). Two types of PRMTs (type I and II) have been identified based on their ability to catalyze the formation of dimethylarginine with type I PRMTs resulting in aDMA and type II PRMTs resulting in sDMA. Both PRMT types are able to cause the formation of MMA intermediates. Currently, eight PRMTs are known in eukaryotes and they are ubiquitously expressed. Glycine and arginine-rich (GAR) regions of proteins are preferred substrates of type I PRMTs, while there are no clear consensus amino acid sequences targeted by type II PRMTs which are able to methylate both isolated arginines as well as arginines within GAR regions. Examples of cellular events affected by arginine methylation include RNA binding and processing, regulation of transcription, signal transduction and DNA repair [18].

Much of the existing knowledge of the importance of protein methylation has been gained through the use of methylation inhibitors which result in the accumulation of proteins in their hypomethylated form. A variety of adenosine analogs have been used to block both protein and RNA methylation. The most commonly used indirect inhibitor of protein methylation is adenosine dialdehyde, also known as adenosine periodate (AdOx) [20-23]. Inhibition of the S-adenosyl-L-homocysteine hydrolase after the addition of AdOx to cells results in the accumulation of S-adenosyl-L-homocysteine which in turn inhibits the action of protein methyltransferase activities [20].

Alterations of protein methylation have been linked to several disease states including idiopathic pulmonary arterial hypertension, hereditary spherocytosis [24], sickle cell anemia [25,26], cancer [27], cardiovascular disease, spinal muscular atrophy, multiple sclerosis, and viral infections [18].

In addition to its involvement in the pathology of diseases, protein methylation has also been shown to be important for virus replication and infectivity in a variety of viruses. Herpes simplex virus (HSV) replication is regulated, in part, by methylation of the RNA binding domain in the HSV ICP27 protein [28]. In vaccinia virus, inhibition of protein methylation resulted in decreased virus replication [29,30]. Protein arginine methylation has also been shown to be required for efficient adenovirus replication [31]. Further, hepatitis delta virus antigen needed to be methylated at arginine residues to support RNA replication [32]. In human immunodeficiency virus (HIV), adenosine analogues have been shown to have anti-viral activity [33]. Interestingly, arginine methylation had a negative impact on the transactivation activity of Tat [34]. PRMT 6 activity was shown to methylate the Tat basic domain *in vitro *and *in vivo *although the precise residues affected are not known. Over-expression of PRMT6 protein in transfected HEK293T and HeLa MAGI cells down regulated Tat-mediated transactivation, while cells treated with siRNA targeting PRMT6 enhanced Tat-mediated transactivation up to approximately 2-fold. Precisely how PRMT6 activity impacts transactivation requires further study.

In this study we investigated the effect of the methylation inhibitor AdOx on the production of HIV-1 in transfected and infected cells as well as on the infectivity of this virus produced in the presence of AdOx. Here we demonstrate increased virus production from transfected or acutely infected cells in the presence of AdOx. However, HIV-1 obtained in this way exhibited defects in Gag-Pol processing, altered morphology, and most importantly a consistent decrease in infectivity. We further outline that the majority of this decreased infectivity is due to the block in HIV-1 entry steps and suggest that methylation of the HIV-1 envelope (Env) protein may influence infectivity, although alternative early events and other viral or cellular proteins could be affected.

## Results

### AdOx treatment results in increased virus production

**Figure 1:**
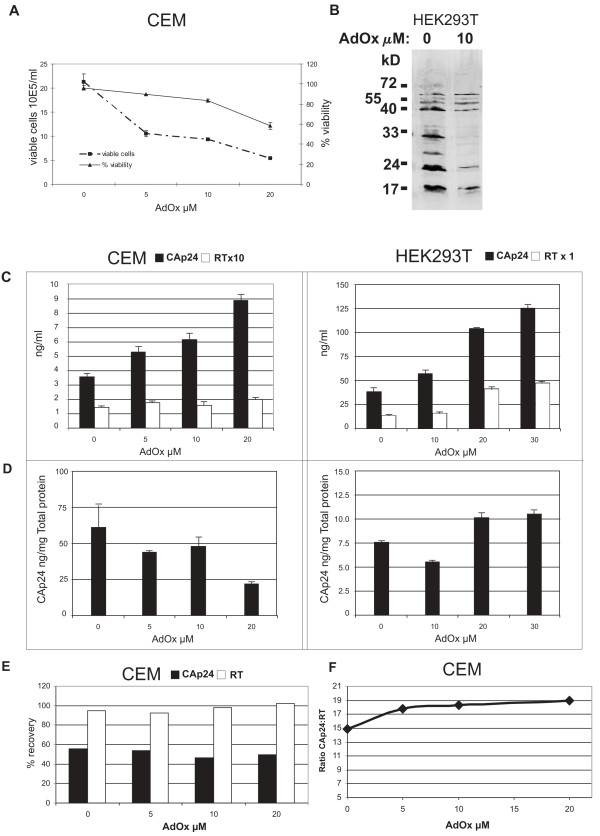
The effect of AdOx on HIV-1 production in CEM T-cells and HEK293T cells. A) CEM cell confluence and viability was assessed using a trypan blue exclusion assay and counting with a hemocytometer. The cell confluence and viability measured in two independent assays and the standard deviation of the mean are shown. B) Western blot of cell lysates from transfected HEK293T cells with the anti-asymmetric dimethylarginine antibody ASYM24 demonstrating a clear reduction in amounts of methylated proteins present in cells treated with 10 μM AdOx compared with cells not treated with AdOx. C) Culture supernatant from the treated CEM cells were assayed for CAp24 and RT content. Shown are representative results from two experiments with the standard deviations of the mean shown. HEK293T cells were transfected with the proviral plasmid pNL4.3 and treated with AdOx as described in the Materials and Methods. Culture supernatant from treated HEK293T cells were assayed for CAp24 and RT content 48 h post transfection. Shown are representative results from two experiments with the standard deviation of the mean shown. D) Whole cell lysates were prepared from infected CEM or transfected HEK293 cells. CAp24 was measured by ELISA of serially diluted lysates and total protein concentration was determined by Bradford assay. Shown is the CAp24 concentration/mg total protein. These experiments were performed from two to four times and the standard deviation of the mean is shown. E) HIV-1 produced by control and AdOx-treated CEM cells were partially purified by ultracentrifugation through a 20% sucrose cushion. The viral pellet was resuspended in RT lysis buffer and the RT and CAp24 levels were measured. The concentration of the initial filtered supernatant, and the amount of residual CAp24 and RT were also measured so that the % recovery could be determined. This experiment was performed twice and a representative result is shown. F) The ratio of the absolute values measured in (E) are shown.

AdOx, an indirect inhibitor of protein methylation, was used in order to determine if this methyltransferase inhibitor affected HIV-1 infectivity. Two cell models were used including HIV-1_NL4.3 _infected CEM cells and HEK293T cells transfected with the HIV-1 proviral plasmid pNL4.3. First, the effect of AdOx on the cell proliferation and viability was determined (Fig. 1A). In the presence of 5 and 10 μM AdOx, CEM cell proliferation was reduced by about half, while effects on cell viability were negligible. However, 20 μM AdOx was moderately toxic to CEM cells compared with the untreated control. HEK293T or HEK293 cells treated with 10–30 μM AdOx displayed no obvious effects on either proliferation or viability (data not shown). However, AdOx concentrations above 30 μM resulted in increased levels of toxicity and loss of cell adherence was observed after prolonged exposure in HEK293T cells. Western analysis using an anti-dimethyl-arginine antibody confirmed that the level of protein methylation was greatly reduced in cells treated with AdOx (Fig. 1B).

The concentration of virus measured as virion capsid p24 protein (CAp24) present in supernatant per 10^5 ^viable CEM cells revealed a marked increase (up to 2.5-fold at the highest AdOx concentration) in CAp24 secretion into culture supernatant with increasing concentrations of AdOx (Fig. 1C). Similarly, CAp24 levels increased in supernatant of transfected HEK293T cells (Fig. 1C). Reverse transcriptase (RT) activity also increased in supernatants from transfected HEK293T. RT levels in culture supernatant collected from CEM cells did not increase proportionate to CAp24, achieving a maximum 1.4-fold increase at 20 μM AdOx (Fig. 1C). We also measured the steady state level of cellular CAp24 in whole cell lysates in either infected CEM or transfected HEK293T cells. Only small changes in CAp24 levels were measured in HEK293T cell lysates, while a 50% decrease in CAp24 was noted in lysates made from infected CEM cells at 20 μM AdOx (Fig. 1D). This result was somewhat surprising given that the amount of secreted CAp24 was substantially increased in CEM cells treated with 20 μM AdOx. Our results are generally consistent with the recent report that demonstrated that HIV virus production is increased after blocking PRMT6 with siRNA [34].

The fact that increased CAp24 was evident in the supernatant compared to whole cell lysates also indicated that AdOx-treatment may have increased virus assembly or budding, or CAp24 secretion. This was examined by purifying AdOx-treated and control virus through a 20% sucrose cushion so that the amount of particulate CAp24 and RT activity could be determined. Overall, the recovery of both proteins was remarkably similar as nearly all of the RT activity and approximately 60% of the total CAp24 was found in pelleted virus (Fig. 1E). However, a distinct change in the relative ratio of CAp24 to RT activity was observed in pelleted virus, increasing from 15 to 19 (ng CAp24:ng RT) in the presence of 20 μM AdOx (Fig. 1F). This was not observed in transfected HEK293T in the presence of 20 μM AdOx suggesting it was a cell type specific effect (data not shown). These results suggest that AdOx treatment may alter either regulation of Gag synthesis or trafficking, assembly at the membrane, or ribosomal frameshifting, which requires further study.

### Biochemical analysis of virus obtained from cells treated with AdOx reveals altered Gag-Pol processing

**Figure 2:**
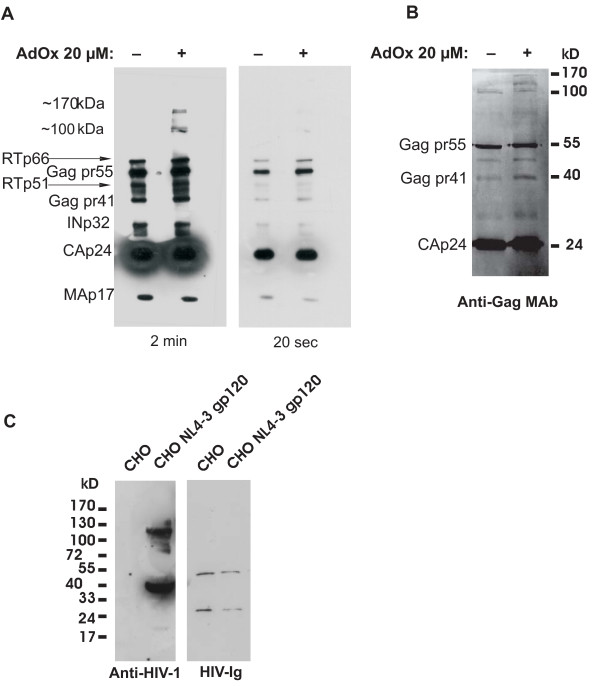
Western blot analysis of partially purified HIV-1 obtained from AdOx-treated cells. A) HIV-1 obtained from AdOx-treated or control CEM T-cells was pelleted through a 20% sucrose cushion. The pelleted virus was solubilized in RT lysis buffer and the CAp24 concentration was determined by ELISA. Western blot analysis was performed using 20 ng of CAp24 and probed with a human HIV-Ig. The proteins detected by the serum were visualized by ECL and two exposures at 2 minutes (left panel) and 20 seconds (right panel) are shown. The short exposure highlights that the amount of total virion protein was equal. The experiment was performed five times with similar results. B) HEK293T cells were treated with 20 μM AdOx 24 h. Equivalent amounts of HIV-1 obtained from cells was purified by centrifugation through 20% sucrose and resuspended in Berman Lysis buffer. After SDS-PAGE, western blot analysis was performed using a monoclonal CAp24 antibody. The results show one of three independent experiments that gave similar results. C) Whole cell lysates prepared from CHO cells or CHO cells stably expressing HIV-1 Env protein subunits gp120 and gp41 were analyzed by Western blot using a goat-anti HIV-1 polyclonal antibody (right panel) or HIV-Ig (left panel).

To determine whether virus produced from infected CEM or transfected HEK293T cells in the presence of AdOx was altered in its composition and either Gag or Gag-Pol processing, viral lysates were separated by SDS-PAGE and Western blotted (Fig. 2). All samples were normalized to either CAp24 or RT. In CEM derived virus, most structural protein and major HIV enzyme levels (RT p66 and integrase) appeared unaltered in virus treated with 20 μM AdOx compared with untreated virus samples (Fig. 2A, CEM derived virus). The viral enzyme levels appeared unaltered in AdOx-treated or control HEK293T cell derived virus in similar Western blots (data not shown). However, a small accumulation of full length or partially processed Gag-Pol precursor was consistently observed in 5 independent CEM-derived and 3 HEK293T-derived virus stocks using either a human anti-HIV immunoglobulin (HIV-Ig) (Fig 2A) or a monoclonal antibody specific for Gag (Fig. 2B).

Finally, Western blot analysis was performed using HIV-Ig or a goat anti-HIV-1 polyclonal antibody to confirm that the 100 to 170 kDa proteins present in the virion lysates obtained from infected CEM cells were not Env (Fig. 2C). Therefore, whole cell lysates were prepared from Chinese hamster ovary (CHO) cells or CHO cells stably expressing high levels of NL4.3 Env. The lysates were probed by Western analysis using procedures identical to those used for the virion lysates (Fig 2A). It was noted that the HIV-Ig antibody did not detect Env under these conditions (Fig. 2C).

Taken together, these results show that AdOx treatment has small effects on Gag-Pol processing resulting in increased amounts of Gag-Pol in virus particles.

### The virus ultrastructure is altered in the presence of AdOx

**Figure 3:**
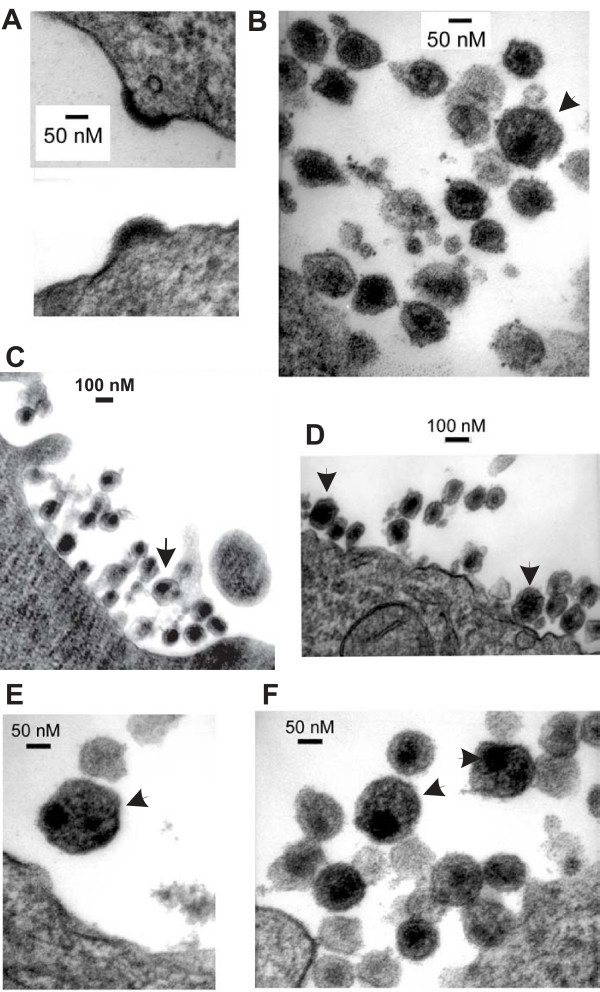
TEM of infected CEM cells. Control (A top panel, B, and C) or AdOx-treated HIV-1 infected CEM (A bottom panel, D E, and F) were thin sectioned and examined by TEM. A) Viewed at 100,000 × showing typical HIV-1 assembly structures. B and C) Viewed 100,000 × and 50,000 ×, respectively, show HIV-1 that are primarily ~100 nm in diameter. A large HIV particle (~150 nm) observed in control sections is indicated by an arrow in B. D) Viewed at 50,000 × shows many typical HIV-1 particles. Two larger virus particles with a diameter of ~150 to ~180 nm are depicted by the arrows. Large HIV-1 particles were present in all sections of AdOx treated cells and two more examples are shown in E and F (both at 100,000 ×). E depicts a particle which appears to contain two core structures.

To further assess what effect AdOx treatment has on virus structure, a cell pellet of day 8 infected CEM cells grown in the presence or absence of 20 μM AdOx for 48 hours was thin-sectioned, epon embedded and assessed by transmission electron microscopy (TEM) (Fig. 3). TEM showed numerous virions in each sample and typical viral assembly structures were observed in both control (Fig. 3A, top panel) and AdOx-treated cells (Fig. 3A, bottom panel). In all sections examined, virus production was only observed in morphologically normal cells. Using the maximum diameter, 191 control virions were measured which had a diameter of 103 ± 13.8 nm, with a characteristic electron dense viral core structure (Fig. 3B and 3C). We measured 223 virions produced by AdOx-treated CEM cells which had a statistically significant larger maximum diameter of 116.6 ± 18.2 nm (p < 10^-10^). Interestingly, we measured 16 virions with a diameter of 140–180 nm (Fig. 3D–F, arrowed) in AdOx-treated samples, while only 3 similarly sized virions were observed in the untreated cell sections (Fig 3B and 3C, arrowed). Larger particles would contain more CAp24 partially explaining why the CAp24:RT ratio increased (Fig 1F), but only if the incorporation of Gag-Pol precursors were not proportionally increased as well. At least one AdOx-treated virion appeared to have two core structures (Fig. 3E). Virus particles that contain two cores have previously been reported to occur as frequent as 33% in MT4 cells [35]. It is possible that methylation of either a viral protein, or a cellular protein, such as a class E vacuolar sorting protein [reviewed in [36]], may be required in order to control the size and the morphology of the assembled virus structure. Further experiments are warranted to determine how AdOx alters the cell milieu resulting in HIV-1 particles of increased size.

### The presence of AdOx during virus production affects viral infectivity

**Figure 4:**
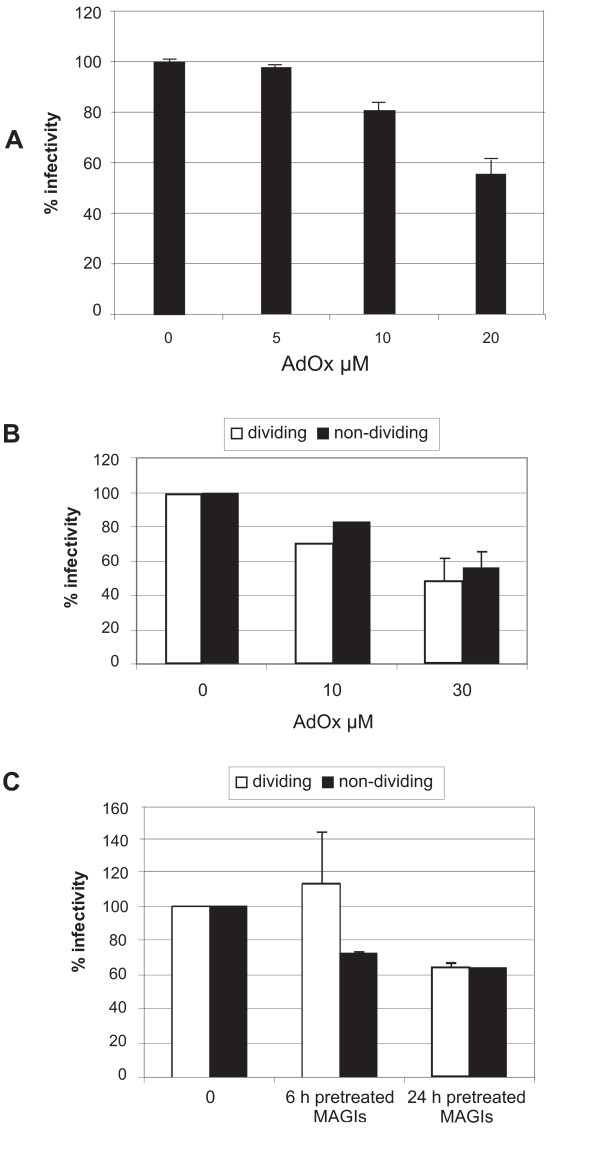
Infectivity of virus produced in the presence of AdOx in a single round replication assay in MAGI-X4 cells. A) MAGI-X4 cells were infected with HIV-1 produced in AdOx-treated CEM cells normalized to 0.1 ng virion associated RT levels. The cells were infected for 2 h after which the virus was removed. After 48 h, the cells were fixed and developed as described in Materials and Methods. The number of blue foci were counted by light microscopy. B) Virus was obtained 48 h post transfection from HEK293T cells treated once with 10 μM AdOx 6 h post transfection. MAGI X4 cells (dividing or γ-irradiated cells) were infected with HIV-1 normalized to RT activity. The cells were infected in a small volume and fresh media was added after 2h without removing virus. After 48 hr, the cells were fixed and developed as described in Materials and Methods. The number of blue foci were counted by light microscopy. C) MAGI-X4 cells were pretreated with a single dose of 10 μM AdOx at 6 or 24 hours before infection with untreated NL4.3 virus. Cells were infected with virus normalized to RT activity. For all experiments, each infection was performed in duplicate. The experiments were performed at least twice using independent virus stocks. The average result and standard deviation of the mean are shown for experiments in A-C.

The multinuclear-activation galactosidase indicator (MAGI) assay using the MAGI-X4 cell line was used to determine the infectivity of virus produced in the presence of AdOx in a single round infectivity assay. When virus produced in infected CEM or transfected HEK293T cells in the presence of AdOx was used to infect MAGI-X4 cells, its infectivity was consistently reduced (Fig. 4A and 4B). The reduction in infectivity was similar in dividing and growth arrested MAGI-X4 cells (Fig. 4B). In growth arrested MAGI-X4 cells, the pre-integration complex (PIC) has to be actively imported into the nucleus in order to allow HIV infection. Hence, the lack of difference in infectivity tends to suggest that PIC nuclear import is not a key factor in the AdOx-induced infectivity changes. To rule out that the observed effects were due to AdOx being present in the virus particle or supernatant, we also performed infections in MAGI-X4 cells pre-treated for 6 or 24 h with a single dose of 10 μM AdOx prior to infection with NL4.3 virus obtained from HEK293T cells without AdOx addition (Fig. 4C). We used this concentration as it greatly exceeded the concentrations present in the small virus inocula which were diluted into normal tissue culture medium. Interestingly, when MAGI-X4 cells were pre-treated 24 hours prior to infection, a reduction of infectivity to 60% of untreated cells was observed in both dividing and growth-arrested cells. This effect was observed irrespective of whether the virus used was obtained in the absence or in the presence of 10 μM AdOx (data not shown), strongly indicating that AdOx present in the virus supernatant does not affect infectivity. These results clearly establish that the effect on infectivity observed with virus obtained from cells in the presence of AdOx is not due to incorporated AdOx which consequently exerts its effect on the MAGI-X4 cells. Another point to note is that infectivity was not altered in dividing cells if MAGI-X4 cells were pre-treated for only 6 h prior to infection, whereas a reduction in infectivity was detectable in growth-arrested cells under the same conditions (Fig. 4C). This suggests that protein methylation of cellular proteins is more important in non-dividing cells and hence a larger effect on infectivity can be observed.

### Vpr overcomes AdOx-induced defects in infectivity in non-dividing cells

**Figure 5:**
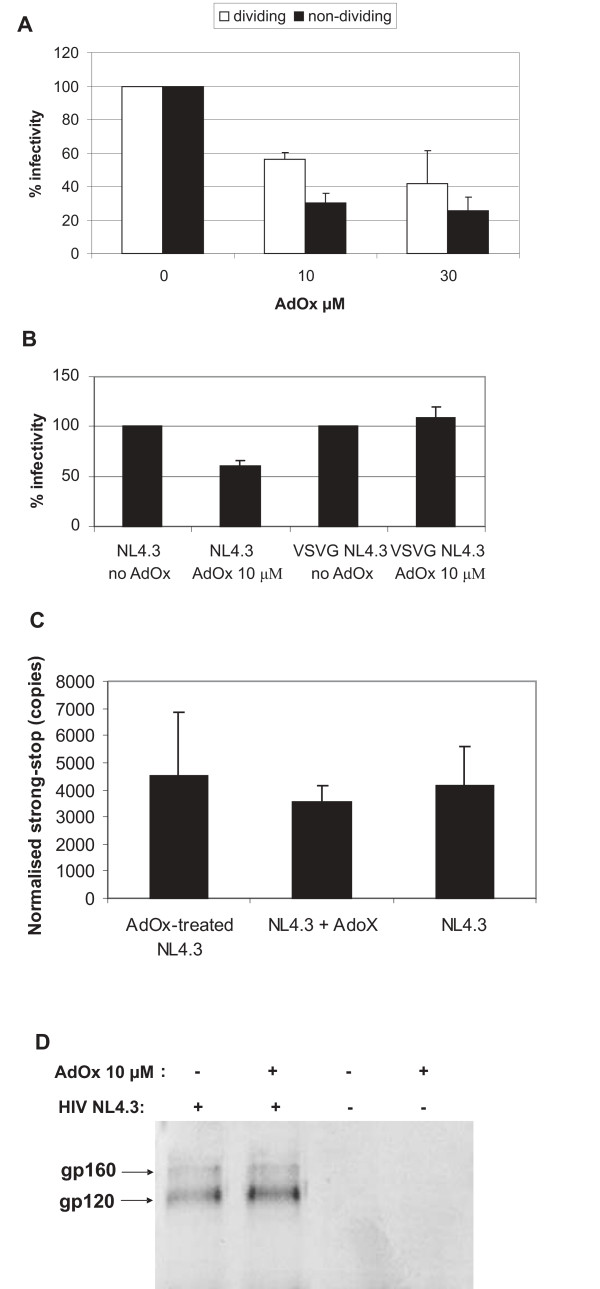
AdOx inhibition acts through Vpr and envelope. A) γ-irradiated or untreated HeLa MAGI-X4 cells were infected with a NL4.3 Vpr-negative HIV molecular clone (pNL4.3VprFS) produced in HEK293T cells treated with AdOx as indicated. Cells were infected with virus normalized to RT activity. Each infection was performed in duplicate. The experiment was performed 5 and 4 times for 10 μM and 30 μM AdOx, respectively, using independent virus stocks. Shown are the average results and the standard deviation of the mean. B) HEK293 cells were transfected with pNL4.3 or pNL4.3*env- *co-transfected with a plasmid expressing VSV-G envelope. HeLa MAGI-X4 cells were infected with equal amounts of each virus normalized to 1 ng virion RT. Shown are the average % infectivity values for three independent experiments and the standard deviation of the mean. The % infectivity value for each AdOx treated virus is shown relative to its respective untreated virus infectivity, with the infectivity of each untreated virus being expressed as 100%. C) HIV-1 infected CEM cells were treated with AdOx as previously described (Fig. 1). ERT reactions were performed using AdOx-treated or control HIV-1. In addition as a control, untreated HIV-1 were supplied exogenous 1 mM AdOx to show that AdOx does not affect ERT. Reverse transcription was initiated by addition of deoxynucleotides and 0.1 mM Triton X-100. The HIV-1 cDNA products were recovered and negative strand strong stop DNA was quantitated by real-time PCR. The copy number indicated was normalized to total RT activity in the virus supernatant. The experiment was performed three times and the standard deviation of the mean is indicated. D) Pelleted virus produced in AdOx -treated or control HEK293T cells were resuspended in Berman lysis buffer. Equal amounts of each virus normalized for RT activity were analyzed by western blot using an anti-gp120 envelope antibody.

The HIV-1 nuclear import protein viral protein R (Vpr) plays a critical role in maintaining infectivity in non-dividing cells [reviewed in [37]]. As our previous results demonstrated no significant differences in virus infectivity in dividing and non-dividing cells in the MAGI-X4 assay (see Fig 4B), we wanted to examine if other viral proteins, in the absence of Vpr, were affected by AdOx resulting in altered infectivity in dividing or non dividing cells. MAGI-X4 cells were infected with NL4.3VprFS virus, which lacks Vpr, obtained from HEK293T cells in the presence of AdOx. It was observed that infectivity was consistently decreased further than observed with NL4.3 wild type virus (compare Fig 5A and 4B). In addition, differences between infectivity in dividing and growth arrested MAGI-X4 cells were more pronounced, with infectivity being more reduced in growth arrested cells compared with dividing cells (Fig 5A). When protein methylation was inhibited by AdOx, infectivity was reduced to a greater extent in non-dividing cells. This suggests that (i) the Vpr protein is not affected by protein methylation, and (ii) that PIC nuclear import in the absence of Vpr is dependent on protein methylation while Vpr-mediated nuclear PIC import is not. Hence, Vpr can partially overcome protein methylation inhibitor induced defects in infectivity of non-dividing cells. In the absence of Vpr, the viral proteins MA and IN are involved in nuclear import of the PIC in non-dividing cells [37] and our data suggest that MA or IN-mediated PIC nuclear import is regulated by protein methylation.

### A pseudotyped envelope relieves the AdOx-induced decrease in virus infectivity

To determine whether AdOx treatment affected HIV-1 entry into MAGI-X4 cells, the infectivity experiment in the MAGI-X4 cell line was repeated with vesicular stomatitis virus G-protein (VSV-G) pseudotyped NL4.3 virus obtained from transfected HEK293 cells. VSV-G pseudotyped virus does not contain HIV envelope glycoproteins and enters cells independent of CD4 and co-receptor interactions via endocytosis. VSV-G pseudotyped and wild type NL4.3 virus stocks were each normalized to RT activity and then used to infect MAGI-X4 cells. Interestingly, VSV-G pseudotyped virus produced in the presence of 10 μM AdOx was as infectious as pseudotyped virus produced in the absence of AdOx, strongly suggesting AdOx-mediated effects occurred via Env, or inhibited an early event that was bypassed via the endosomal entry pathway (Fig. 5B). In these experiments, we noted that 10 μM AdOx consistently inhibited infectivity by 40% whereas the same AdOx concentration in a previous experiment inhibited infectivity by ~20%. Irrespective of this difference, the results here agree that AdOx can reduce HIV-1 infectivity, but can be bypassed if entry is mediated via the endosomal pathway. This implies that AdOx treatment results in a block somewhere between receptor binding or viral uncoating.

Endogenous reverse transcription (ERT) reactions were performed to determine if virions had a defect in the ability to initiate DNA synthesis (Fig 5C). Three virus preparations were compared: HIV-1 NL4.3 made by HEK293T cells treated with 10 μM AdOx (final concentration of 1 μM in the ERT reaction), untreated HIV-1 NL4.3 supplemented with 1 μM AdOx, or untreated HIV-1 NL4.3 without AdOx. The ERT was initiated by the addition of 0.1 mM Trition-X-100 and deoxynucleotides, and the cDNA products were measured by quantitative PCR using oligonucleotides specific for a HIV-1 negative strand strong-stop DNA, the first product of reverse transcription. No statistically significant differences were observed in the ability to initiate DNA synthesis in AdOx-treated or control HIV-1 (p = 0.83) (Fig. 5C). It is highly unlikely that a reverse transcription defect is responsible for the decreased infectivity induced by AdOx treatment.

To determine whether AdOx affected the amount of envelope glycoprotein incorporated into virus particles in the producer cells, viral lysates produced in HEK293T cells in the presence or absence of a single addition of 10 μM AdOx were analyzed by Western blot using a monoclonal gp120 antibody. No significant differences in the amount of glycoprotein incorporated into virus particles were observed in virus produced from HEK293T cells (Fig 5D) or HEK293 cells (data not shown), suggesting that AdOx treatment may inhibit an early entry step such as receptor binding or fusion rather than the incorporation of Env into virions.

## Discussion

It is becoming increasingly apparent that protein methylation is particularly important in many key cellular events and recent studies have suggested that viral replication can be regulated by protein methylation [6,29,30,33,34]. In particular, HIV-1 replication has been shown to be inhibited by adenosine analogues suggesting an important role for protein methylation [33]. More recently, it was shown that the HIV-1 Tat protein can be methylated on arginine residues by PRMT6 [34]. In their study, Boulanger et al. specifically reduced cellular levels of PRMT6 using siRNA which resulted in Tat arginine methylation inhibition and consequently increased virus production from HEK293T cells. Here, the non-specific methylation inhibitor AdOx was used to globally inhibit protein methylation in cells during the production of virus in two different systems, either in transfected HEK293T cells or in infected CEM cells. Consistent with findings by Boulanger et al., we also detected an increase in the amount of virus produced in both systems of virus production (Fig 1), indicating that blocking protein methylation in virus producing cells results in an increase in virus output in two different cell types and in both infected as well as transfected cells. The major focus of this study was to determine whether protein methylation (other than Tat) was important for virus infectivity and involved the further characterization of virus produced in cells when protein methylation was inhibited by AdOx.

Firstly, it was demonstrated that the major structural proteins and enzymes were present in virus produced in CEM cells when protein methylation was inhibited by AdOx (Fig. 2). In addition, we detected an increase in the relative ratio of CAp24 to RT in virus produced in AdOx-treated CEM cells indicating that protein methylation affected virus assembly. Curiously this was not observed in HEK293T cells suggesting this effect was cell type dependent. Secondly, there were detectable differences in virus protein composition detected between virus produced in cells in the presence or absence of AdOx where an increase of Gag-Pol and partially processed intermediates in CEM and in HEK293T cells were observed by Western blot (Fig. 2). The increase in the amount of Gag-Pol precursor in both CEM and HEK293T cells suggests that a defect in the cleavage of the Gag-Pol intermediate may be affected by protein methylation. Irrespective of the exact mechanism and cause for the observed alterations in Gag-Pol processing in the presence of AdOx, it is unlikely that these subtle differences detected in either of the cell lines are the main reason for the observed effects on virus infectivity (Fig. 4).

Curiously, TEM showed that AdOx treated HIV-1-infected CEM cells made larger particles indicating that a methylation pathway somehow contributes to viral particle formation. It was recently estimated that an increase in virion diameter from 119 nm to 207 nm increased CAp24 content from 3,000 to 11,000 molecules [39]. Using similar calculations, we estimate that approximately 1800 CAp24 molecules were present in a control virion and approximately 2900 CAp24 molecules in an AdOx-treated virion. This may at least partially account for the increased ratio of CAp24 to RT measured in AdOx treated virions. Why RT levels were relatively lower compared to CAp24 in CEM cells is not clear but possible explanations include that AdOx may alter RT frame shifting or RT trafficking to assembling virions. It is not clear how protein methylation could impact on virion size and whether this is due to protein methylation of a viral or cellular protein, or an altered cell transcriptome.

Despite the increase in virus production in the presence of AdOx from both cell types (see Fig. 1), virus produced in the presence of AdOx displayed altered infectivity in a single round replication assay (Fig. 4). The effect on infectivity was dependent on the concentration of AdOx present during virus production, with reduced infectivity at higher AdOx concentrations in both dividing and growth arrested cells. This effect was observed irrespective of the cell type used to produce the virus. Although AdOx was moderately toxic to CEM cells at 20 μM and not toxic to 293T cells, virus biochemistry was only modestly affected in both cell types (increased levels of CAp24 in CEM, low amounts of unprocessed Gag-Pol in both cell types) clearly separating cellular toxicity from virion composition defects. It was noted during the TEM examination of all cell sections that virus production was only observed in morphologically healthy looking cells, and not in apoptotic or necrotic cells. We ruled out that these effects were due to AdOx being incorporated into virions and exerting its effect on the infected MAGI-X4 cells, by exposing MAGI-X4 cells to AdOx prior to infection with non-treated virus. Moreover, AdOx does not block methylation directly but inhibits synthesis of the important precursor molecule homocysteine. It is extremely unlikely that sufficient AdOx could be present in the small virus inocula to deplete the endogenous pools of S-adenosyl methionine in the MAGI-X4 cells. These results strongly suggest that protein methylation of one or more of the proteins present in the virus particle can regulate virus infectivity.

Interestingly, the cellular factor Sam68 can bind to the Rev response element of HIV-1 and functionally replace or synergize with Rev to regulate the transport of unspliced HIV-1 mRNA [40-42]. Sam-like proteins, including SLM-1, SLM2, and others, also have the ability to replace Rev function [reviewed in [43]]. Recently, it was shown that PRMT1 uses Sam68 as a substrate leading to asymmetric dimethylarginine formation [44]. Methylation was required for Sam68 function and inhibition with 250 μM AdOx resulted in cytoplasmic accumulation of Sam68 and decreased transport of unspliced HIV-1 mRNA. While 10–20 μM AdOx is sufficient to generate hypomethylated proteins in cells (1) [see [21,45]], many studies use much higher concentrations (0.1–1 mM). We avoided very high levels of AdOx as they have been shown to be associated with defects in cell cycle progression, cell apoptosis, and necrosis [46], and resulted in cell toxicity.

In an attempt to identify which viral proteins (other than Tat) were involved in the observed effects on HIV-1 infectivity after addition of AdOx, the HIV-1 viral mutant NL4.3VprFS was used. This virus lacks a functional *vpr *gene due to a frameshift mutation at amino acid 63 in the *vpr *open reading frame. The accessory viral protein Vpr has been shown to be involved in viral transactivation by acting as a transcriptional activator [47], in the nuclear import of the pre-integration complex [37] allowing replication of HIV-1 in non-dividing cells [48], as well as in other activities like regulation of apoptosis and cell cycle arrest [49-53]. Because protein methylation has been described in several of these cellular events, most importantly the regulation of transcription [6-8] and nuclear transport [11], and Vpr possesses two predicted methylation sites (at amino acid R84 and R87) and is incorporated into virions, it is a likely candidate to be involved in the methylation-dependent regulation of infectivity. If Vpr itself needs to be methylated in order to maintain sufficient infectivity, then infectivity of virus lacking Vpr (NL4.3VprFS) should not be affected by the presence of AdOx. Interestingly, our infectivity results with NL4.3VprFS virus produced in the presence of AdOx indicate that the observed decreases in infectivity were due to inhibition of a protein other than Vpr. In the absence of Vpr, decreases in infectivity after AdOx treatment were more pronounced than in virus containing Vpr. In addition, differences between infectivity in dividing and growth arrested MAGI-X4 cells were more pronounced in the absence of Vpr. A differential effect on infectivity in dividing and growth arrested MAGI-X4 cells suggests an effect on PIC nuclear transport. Vpr, MA and IN have been suggested to be involved in PIC nuclear transport possibly in a redundant fashion [37]. One possible explanation for the differential infectivity in dividing and growth arrested MAGI-X4 cells in the absence of Vpr could be that one of the other proteins that can compensate for Vpr function in terms of PIC transport and/or nuclear import needs to be methylated in order to fulfill this function. In the absence of Vpr in NL4.3VprFS virus and in the absence of sufficient protein methylation, a compounding decrease in infectivity in particular in growth arrested cells, as observed in this study, would be the result. These data further support that multiple, complex and possibly opposing and/or redundant mechanisms of protein methylation are likely involved in regulating HIV-1 infectivity.

We also determined the infectivity of virus lacking the HIV-1 envelope glycoprotein using VSV-G pseudotyped virus produced in HEK293 cells in the presence of AdOx. Importantly, we demonstrated that the decreased infectivity in the presence of AdOx was completely restored to wild type levels when HIV-1 Env was replaced by VSV-G. One possibility is that HIV-1 Env methylation might contribute towards virus infectivity, although it is equally plausible that an endosomal entry pathway bypassed an early event which was inhibited in virus made by AdOx-treated cells. However others have reported infectivity defects restored by pseudotyping. For example, an HIV-1 construct containing multiple mutations in MA had reduced infectivity in MAGI cells, but infectivity could be restored by pseudotyping HIV-1 with VSV-G [54]. Given an ERT assay failed to measure significant defects in reverse transcription, it is distinctly possible there is a defect somewhere between receptor binding and uncoating. Deciphering the precise event affected requires further experiments. In either case, our Western blot data clearly show that the reduced infectivity after AdOx treatment was not due to decreased envelope glycoprotein incorporation into virions, suggesting that the defect is in the early receptor binding and/or an early entry event. A prediction of methylation sites within gp160 of NL4.3 using MeMo identified 10 arginine residues and 28 lysine residues [57]. Work in our laboratories is currently focusing on determining which steps of early replication are affected, if residues in Env are the target by PRMT activity, and if this accounts for reduced infectivity induced by AdOx treatment on virus producing cells.

## Conclusion

Overall, we have shown that protein methylation contributes towards maximal virus infectivity. Furthermore, our results also indicate that protein methylation regulates HIV-1 infectivity in a complex manner most likely involving the methylation of multiple viral or cellular proteins and/or multiple steps of replication. With the increased knowledge of the importance of protein methylation in a wide range of cellular events, it is important to fully determine the exact role and mechanism of both viral protein methylation and methylation of cellular proteins involved in virus replication. This information will be essential in order to investigate whether regulation of protein methylation may be considered a potential target for future novel antivirals in the combat of HIV-1 and possibly other viral infections.

## Methods

### Cell lines

CEM cells (acute lymphoblastic T-cell leukaemic cells) were maintained in Dulbeccos RPMI 1640 supplemented with 10% FBS, 100 U/ml penicillin and 0.1 mg/ml streptomycin. HEK 293T cells (human embryonic kidney cells), HEK293, and the HeLa derived MAGI-X4 cell lines were maintained in DMEM containing 10% FCS, 100 U/ml penicillin, 0.1 mg/ml streptomycin and 2 mM L-glutamine. Media for the MAGI-X4 cells also contained G418 (0.2 mg/ml), hygromycin B (0.1 mg/ml) and puromycin (1 μg/ml). Chinese hamster ovary cells (CHO) and CHO stably expressing ENV, kindly provided by Andreas Suhrbier, were cultured in RPMI 1640 supplemented with 10% FBS, 100 U/ml penicillin and 0.1 mg/ml streptomycin

### Virus production, purification and quantitation

The molecular cloned HIV-1 virus pNL4.3 and a frameshift mutant of pNL4.3 resulting in a Vpr-negative HIV-1 variant (pNL4.3VprFS) were used in this study. Virus was obtained either from HEK293T cells transfected with 9 μg maxi-prep DNA (QIAGEN) using lipofectamine 2000 (Invitrogen) or from infected CEM cells. For pseudotyped HIV-1, HEK293 cells were transfected with 6.5 μg pNL4.3.LUC.RE and 1.5 μg of pHEF-VSVG (Both NIH Reference and Reagents Program), or 8 μg of pNL4.3. The amount of virus in filtered, infectious supernatants was measured using either a CAp24 ELISA (Zeptometrix), a functional reverse transcriptase (RT) assay (RT Detect from Roche) or an in-house RT assay as previously described [55,56]. Virus was pelleted either through a 20% sucrose cushion via ultracentrifugation at 100,000 × *g *for 2 h at 4°C, or at 20,000 × g for 2 h at 4°C. Cell lysates were prepared from both infected CEM and transfected HEK293T cells. The former were lysed in 1X RIPA buffer (50 mM Tris-HCl (pH 8.0), 150 mM NaCl, containing 1% TritonX-100, 0.1% SDS, 0.5% sodium deoxycholate, 2 μg/ml pepstatin A and 1 tablet of Complete, EDTA- free protease inhibitor cocktail tablets/50 ml buffer (Roche), and the later in 1X PBS containing 1% Triton-X100 and protease inhibitor cocktail.

### Western blotting

Pelleted virus was lysed in either RT lysis buffer (50 mM Tris, 80 mM potassium chloride, 2.5 mM DTT, 0.75 mM EDTA, 0.5% Triton X-100, pH7.8) or in Berman lysis buffer (0.1% SDS, 0.5% sodium deoxycholate, 1% NP-40 in PBS) containing 1 tablet of complete protease inhibitor tablet (Roche) per 10 ml, and CAp24 content and RT activity were measured as described above. An equivalent amount of virus normalized for Cap24 or RT was then separated on SDS-PAGE before transferring to PVDF membranes. Western blots were developed using a monoclonal CAp24 antibody, a polyclonal HIV-Ig or a monoclonal gp120 antibody (cat # 4121, 3957 from the NIH Reference and Reagents Program and cat# ARP3119 from Ms C Arnold provided through the NIBSC centralized facility for AIDS Reagents, supported by the EU Programme EVA and the UK Medical Research Council, respectively).

Cell lysates were prepared from CHO, or CHO-ENV cells using cell RIPA lysis buffer. The protein concentration was determined by a bradford assay, and 20 μg of protein was separated by SDS-PAGE on a 4–20% gradient polyacrylamide gel. The protein was transferred to a PVDF membrane and probed using an HIV-Ig at 1:2000 dilution, or a goat anti-HIV-1 polyclonal antibody (BioDesign) at a 1:1000 dilution. The HEK293T lysates were prepared with Berman lysis buffer and similarly probed using an anti-dimethyl-arginine antibody (ASYM24, Upstate) at a 1:1000 dilution. Each antibody was detected using an appropriate horse radish peroxidase (HRP) or Alkaline Phosphatase-conjugated secondary antibody and SuperSignal West Pico chemiluminescent substrate (Pierce) or BCIP/NBT substrate (Sigma).

### Infections in CEM cells

For virus production from CEM cells, ~1.3 × 10^5 ^CEM cells/ml were co-cultured with ~5 × 10^4 ^cells/ml chronically HIV-1 infected CEM cells. Virus production was monitored visually for cytopathic effects and by CAp24 ELISA. AdOx experiments were performed 8 days post co-culture.

### Treatment with AdOx

Adenosine periodate (Sigma) was solubilized at 1 or 15 mM stock concentration in milliQ H_2_O, stored frozen and added at various times to the cultured cells at the indicated final concentrations. For assessment of CEM viability in the presence of AdOx, 7 × 10^5 ^CEM cells/ml were grown in the presence of various concentrations of AdOx for 24 hours. Thereafter, the media was replaced with fresh media containing AdOx and the cells were grown for a further 24 hours prior to assessing viability using a trypan blue exclusion assay and a hemocytometer. Treated and untreated virus supernatant was collected and passed through a 0.45 μm filter. For virus production from transfected HEK293T cells, AdOx was initially added 4–6 hours after transfection. Thereafter, the media and AdOx were replaced 24 h post transfection and viral supernatants were collected at 48 hours post transfection and filtered through 0.45 μm filters.

### Infectivity assay

The single round infectivity assay in the MAGI-X4 cell line was used to compare infectivity of virus produced from infected CEM and transfected HEK293T or HEK293 cells in the presence or absence of AdOx. MAGI-X4 cells were seeded in 6 or 24 well plates one day prior to infection. Virus normalized to RT was added in a total volume of 200–500 μL for 2–4 h, and cells were grown for an additional 48 hours before fixing and staining with X-gal for β-galactosidase activity. Blue foci, indicative of infected cells were counted under a light microscope. MAGI-X4 cells were infected with virus obtained in the presence or absence of AdOx from CEM, HEK293T, or HEK293 cells. This infectivity assay was also conducted in MAGI-X4 cells pre-treated with AdOx at a single dose of 10 μM either 6 h or 24 h prior to infection with virus obtained from cells not treated with AdOx. Infectivity in growth arrested (via γ-irradiation at 6000 Rad) MAGI-X4 cells was also assessed using the same virus inocula as before.

### Transmission electron microscopy

To assess the effect of AdOx on HIV-1 infected CEM cells by Transmission Electron Microscopy (TEM), the cells (7 × 10^5 ^cells/ml) were grown in the presence or absence of 20 μM AdOx for 24 hours. Thereafter, the media was replaced with fresh media containing AdOx and the cells were grown for a further 24 hours. These treatments were performed in parallel with the CEM AdOx treatments described above. The cells were harvested by centrifugation, washed once with PBS, then each pellet was resuspended in 2 ml of 3% glutaraldehyde in 0.1 M sodium phosphate buffer (pH 7.4) and incubated at room temperature for 2 h. The cells were washed with 0.1 M sodium phosphate buffer and treated with 1% osmium tetroxide in 0.1 M sodium cacodylate buffer for 40 minutes at room temperature. The cells were next washed with 0.1 M sodium cacodylate buffer, followed by 80% acetone and stained with 2% uranyl acetate in 80% acetone overnight at 4°C. The pellets were then dehydrated, embedded in epon resin and polymerized at 65°C. 100 nm thick sections were cut and EM grids (on 200 mesh copper) (ProSciTech) were prepared for each sample. Each grid (containing 4–5 sections) was stained with 5% uranyl acetate, followed by lead citrate, rinsed with distilled water and then visualized by TEM. The staining, epon embedding, ultramicrotomy and TEM analysis were performed by Deborah Stenzel at the Analytical Electron Microscopy Facility, The Queensland University of Technology.

### ERT assay

Reverse transcription products were generated by addition of virus particles to a mixture (final volume of 50 μl) containing 10 mM Tris, pH 7.4, 10 mM MgCl_2_, 500 U/ml DNase I (unless otherwise indicated) and 200 μM of each dNTP in RPMI1640 medium for up to 20 h as indicated. Triton X-100 and AdOx were included at the concentrations indicated in the text. A no nucleotide control reaction was always included. Products were extracted, once with an equal phenol:chloroform:iso-amyl alcohol (25:24:1) and once with chloroform. The extracts were ethanol precipitated, washed with 70% ethanol, dried and resuspended in 100 μl of 0.1 mM EDTA. Purified reaction products (5 μl) were added to the reaction mix containing 0.4 μM of each primer, SYBR Green I, 30 U/ml Platinum Taq polymerase, 20 mM Tris-HCl pH 8.4, 50 mM KCl, 3 mM MgCl_2_, 200 μM each dNTP, 20 U/l uracil-N-glycosylase (Invitrogen, Carlsbad, CA, USA) in a final volume of 15 μl. A no-DNA control (5 μl of 0.1 mM EDTA, pH 8.0) was also included. Standard primer sets used for amplification were strong-stop DNA reverse (5'-d AAGCAGTGGGTTCCCTAGTTAG-3') and forward (5'-d GGTCTCTCTGGTTAGACCA-3') oligonucleotides. The mixes were subjected to cycling: [50°C, 2 min; 95°C, 2 min]_1 _[95°C, 15 sec; 65°C, 30 sec]_40 _on a Rotor-Gene 3000™ thermocycler (Corbett) set to collect SYBR fluorescent signal after the 65°C step. Copy number was determined by reference to a standard curve prepared by dilution of plasmid DNA (NL4.3 strain). A no-nucleotide control was always included and was negligible or the data were discarded. Total RT activity in each virus supernatant was measured using the Roche RT Detect assay and this value was used to normalize measured DNA copies.

## Competing interests

The author(s) declare they have no competing interests.

## Authors' contributions

NMW and EMH contributed equally and performed the majority of the experiments described in the manuscript, analyzed results, and prepared a draft of the manuscript. TJB and AA made virus stocks and performed western blot experiments. DW produced the pseudotype virus stocks and assisted with experiments. SCP and DH jointly initiated, designed and co-supervised this project, analyzed results and wrote the manuscript.

## References

[B1] Aletta JM, Cimato TR, Ettinger MJ (1998). Protein methylation: a signal event in post-translational modification. Trends in Biochemical Sciences.

[B2] Clarke S (1993). Protein methylation. Current Opinion in Cell Biology.

[B3] Wang Y, Wysocka J, Sayegh J, Lee YH, Perlin JR, Leonelli L, Sonbuchner LS, McDonald CH, Cook RG, Dou Y, Roeder RG, Clarke S, Stallcup MR, Allis CD, Coonrod SA (2004). Human PAD4 regulates histone arginine methylation levels via demethylimination. Science.

[B4] Cuthbert GL, Daujat S, Snowden AW, Erdjument-Bromage H, Hagiwara T, Yamada M, Schneider R, Gregory PD, Tempst P, Bannister AJ, Kouzarides T (2004). Histone deimination antagonizes arginine methylation. Cell.

[B5] Zhang J, Dai J, Zhao E, Lin Y, Zeng L, Chen J, Zheng H, Wang Y, Li X, Ying K, Xie Y, Mao Y (2004). cDNA cloning, gene organization and expression analysis of human peptidylarginine deiminase type VI. Acta Biochim Pol.

[B6] Lee DY, Teyssier C, Strahl BD, Stallcup MR (2005). Role of protein methylation in regulation of transcription. Endocrine Reviews.

[B7] Stallcup MR (2001). Role of protein methylation in chromatin remodeling and transcriptional regulation. Oncogene.

[B8] Stallcup MR, Kim JH, Teyssier C, Lee YH, Ma H, Chen D (2003). The roles of protein-protein interactions and protein methylation in transcriptional activation by nuclear receptors and their coactivators. Journal of Steroid Biochemistry & Molecular Biology.

[B9] Clarke S (2003). Aging as war between chemical and biochemical processes: protein methylation and the recognition of age-damaged proteins for repair. Ageing Research Reviews.

[B10] Blanchet F, Cardona A, Letimier FA, Hershfield MS, Acuto O (2005). CD28 costimulatory signal induces protein arginine methylation in T cells. Journal of Experimental Medicine.

[B11] Smith WA, Schurter BT, Wong-Staal F, David M (2004). Arginine methylation of RNA helicase a determines its subcellular localization. Journal of Biological Chemistry.

[B12] Kujubu DA, Stimmel JB, Law RE, Herschman HR, Clarke S (1993). Early responses of PC-12 cells to NGF and EGF: effect of K252a and 5'-methylthioadenosine on gene expression and membrane protein methylation. Journal of Neuroscience Research.

[B13] Cimato TR, Ettinger MJ, Zhou X, Aletta JM (1997). Nerve growth factor-specific regulation of protein methylation during neuronal differentiation of PC12 cells. Journal of Cell Biology.

[B14] Vemuri R, Philipson KD (1988). Protein methylation inhibits Na+-Ca2+ exchange activity in cardiac sarcolemmal vesicles. Biochimica et Biophysica Acta.

[B15] Chen YF, Zhang AY, Zou AP, Campbell WB, Li PL (2004). Protein methylation activates reconstituted ryanodine receptor-ca release channels from coronary artery myocytes. Journal of Vascular Research.

[B16] Mowen KA, Schurter BT, Fathman JW, David M, Glimcher LH (2004). Arginine methylation of NIP45 modulates cytokine gene expression in effector T lymphocytes.[see comment]. Molecular Cell.

[B17] McBride AE, Silver PA (2001). State of the arg: protein methylation at arginine comes of age. Cell.

[B18] Bedford MT, Richard S (2005). Arginine methylation an emerging regulator of protein function. Molecular Cell.

[B19] Gary JD, Clarke S (1998). RNA and protein interactions modulated by protein arginine methylation. Prog Nucleic Acid Res Mol Biol.

[B20] Johnson BA, Najbauer J, Aswad DW (1993). Accumulation of substrates for protein L-isoaspartyl methyltransferase in adenosine dialdehyde-treated PC12 cells. Journal of Biological Chemistry.

[B21] Chen DH, Wu KT, Hung CJ, Hsieh M, Li C (2004). Effects of adenosine dialdehyde treatment on in vitro and in vivo stable protein methylation in HeLa cells. J Biochem (Tokyo).

[B22] Li C, Ai LS, Lin CH, Hsieh M, Li YC, Li SY (1998). Protein N-arginine methylation in adenosine dialdehyde-treated lymphoblastoid cells. Archives of Biochemistry & Biophysics.

[B23] Najbauer J, Aswad DW (1990). Diversity of methyl acceptor proteins in rat pheochromocytoma (PC12) cells revealed after treatment with adenosine dialdehyde. Journal of Biological Chemistry.

[B24] Ingrosso D, D'Angelo S, Perna AF, Iolascon A, Miraglia del Giudice E, Perrotta S, Zappia V, Galletti P (1995). Increased membrane-protein methylation in hereditary spherocytosis. A marker of cytoskeletal disarray. European Journal of Biochemistry.

[B25] Manna C, Hermanowicz N, Ro JY, Neilan B, Glushko V, Kim S (1984). Abnormal membrane protein methylation and merocyanine 540 fluorescence in sickle erythrocyte membranes. Biochemical Medicine.

[B26] Ro JY, Neilan B, Magee PN, Paik WK, Kim S (1981). Reduced erythrocyte membrane protein methylation in sickle cell anemia. Journal of Biological Chemistry.

[B27] Duerre JA, DiMaria P, Kim S, Paik WK (1991). Current status of protein methylation in carcinogenesis. Critical Reviews in Oncogenesis.

[B28] Mears WE, Rice SA (1996). The RGG box motif of the herpes simplex virus ICP27 protein mediates an RNA-binding activity and determines in vivo methylation. Journal of Virology.

[B29] Borchardt RT, Keller BT, Patel-Thombre U (1984). Neplanocin A. A potent inhibitor of S-adenosylhomocysteine hydrolase and of vaccinia virus multiplication in mouse L929 cells. Journal of Biological Chemistry.

[B30] Keller BT, Borchardt RT (1987). Adenosine dialdehyde: a potent inhibitor of vaccinia virus multiplication in mouse L929 cells. Molecular Pharmacology.

[B31] Kzhyshkowska J, Kremmer E, Hofmann M, Wolf H, Dobner T (2004). Protein arginine methylation during lytic adenovirus infection. Biochemical Journal.

[B32] Li YJ, Stallcup MR, Lai MM (2004). Hepatitis delta virus antigen is methylated at arginine residues, and methylation regulates subcellular localization and RNA replication. Journal of Virology.

[B33] Gordon RK, Ginalski K, Rudnicki WR, Rychlewski L, Pankaskie MC, Bujnicki JM, Chiang PK (2003). Anti-HIV-1 activity of 3-deaza-adenosine analogs. Inhibition of S-adenosylhomocysteine hydrolase and nucleotide congeners. European Journal of Biochemistry.

[B34] Boulanger MC, Liang C, Russell RS, Lin R, Bedford MT, Wainberg MA, Richard S (2005). Methylation of Tat by PRMT6 regulates human immunodeficiency virus type 1 gene expression. Journal of Virology.

[B35] Briggs JA, Wilk T, Welker R, Krausslich HG, Fuller SD (2003). Structural organization of authentic, mature HIV-1 virions and cores. Embo J.

[B36] Morita E, Sundquist WI (2004). Retrovirus budding. Annu Rev Cell Dev Biol.

[B37] Piller SC, Caly L, Jans DA (2003). Nuclear import of the pre-integration complex (PIC): the Achilles heel of HIV?. Curr Drug Targets.

[B38] Stark LA, Hay RT (1998). Human immunodeficiency virus type 1 (HIV-1) viral protein R (Vpr) interacts with Lys-tRNA synthetase: implications for priming of HIV-1 reverse transcription. J Virol.

[B39] Briggs JA, Simon MN, Gross I, Krausslich HG, Fuller SD, Vogt VM, Johnson MC (2004). The stoichiometry of Gag protein in HIV-1. Nat Struct Mol Biol.

[B40] Reddy TR, Xu W, Mau JK, Goodwin CD, Suhasini M, Tang H, Frimpong K, Rose DW, Wong-Staal F (1999). Inhibition of HIV replication by dominant negative mutants of Sam68, a functional homolog of HIV-1 Rev. Nat Med.

[B41] Soros VB, Carvajal HV, Richard S, Cochrane AW (2001). Inhibition of human immunodeficiency virus type 1 Rev function by a dominant-negative mutant of Sam68 through sequestration of unspliced RNA at perinuclear bundles. J Virol.

[B42] Modem S, Badri KR, Holland TC, Reddy TR (2005). Sam68 is absolutely required for Rev function and HIV-1 production. Nucleic Acids Res.

[B43] Lukong KE, Richard S (2003). Sam68, the KH domain-containing superSTAR. Biochim Biophys Acta.

[B44] Cote J, Boisvert FM, Boulanger MC, Bedford MT, Richard S (2003). Sam68 RNA binding protein is an in vivo substrate for protein arginine N-methyltransferase 1. Mol Biol Cell.

[B45] Belyanskaya LL, Delattre O, Gehring H (2003). Expression and subcellular localization of Ewing sarcoma (EWS) protein is affected by the methylation process. Exp Cell Res.

[B46] Schwerk C, Schulze-Osthoff K (2005). Methyltransferase inhibition induces p53-dependent apoptosis and a novel form of cell death. Oncogene.

[B47] Kino T, Gragerov A, Slobodskaya O, Tsopanomichalou M, Chrousos GP, Pavlakis GN (2002). Human immunodeficiency virus type 1 (HIV-1) accessory protein Vpr induces transcription of the HIV-1 and glucocorticoid-responsive promoters by binding directly to p300/CBP coactivators. J Virol.

[B48] Connor RI, Chen BK, Choe S, Landau NR (1995). Vpr is required for efficient replication of human immunodeficiency virus type-1 in mononuclear phagocytes. Virology.

[B49] Emerman M (1996). HIV-1, Vpr and the cell cycle. Curr Biol.

[B50] Fukumori T, Akari H, Yoshida A, Fujita M, Koyama AH, Kagawa S, Adachi A (2000). Regulation of cell cycle and apoptosis by human immunodeficiency virus type 1 Vpr. Microbes Infect.

[B51] Gil J, Bermejo M, Alcami J (2004). HIV and apoptosis: a complex interaction between cell death and virus survival. Prog Mol Subcell Biol.

[B52] Hrimech M, Yao XJ, Branton PE, Cohen EA (2000). Human immunodeficiency virus type 1 Vpr-mediated G(2) cell cycle arrest: Vpr interferes with cell cycle signaling cascades by interacting with the B subunit of serine/threonine protein phosphatase 2A. Embo J.

[B53] Waldhuber MG, Bateson M, Tan J, Greenway AL, McPhee DA (2003). Studies with GFP-Vpr fusion proteins: induction of apoptosis but ablation of cell-cycle arrest despite nuclear membrane or nuclear localization. Virology.

[B54] Kaushik R, Ratner L (2004). Role of human immunodeficiency virus type 1 matrix phosphorylation in an early postentry step of virus replication. J Virol.

[B55] Suzuki K, Saito T, Kondo M, Osanai M, Watanabe S, Kano T, Kano K, Imai M (1995). Poly A-linked non-isotopic microtiter plate reverse transcriptase assay for sensitive detection of clinical human immunodeficiency virus isolates. J Virol Methods.

[B56] Suzuki K, Craddock BP, Okamoto N, Kano T, Steigbigel RT (1993). Poly A-linked colorimetric microtiter plate assay for HIV reverse transcriptase. J Virol Methods.

[B57] Methylation Modification Prediction Server (2.0). http://www.bioinfo.tsinghua.edu.cn/~tigerchen/memo.html.

